# Structural analyses of antigen binding similarities and differences between rabbit and human anti-gp120 V3 mAbs

**DOI:** 10.1186/1742-4690-9-S2-P76

**Published:** 2012-09-13

**Authors:** R Pan, JM Sampson, Y Chen, M Vaine, S Wang, S Lu, X Kong

**Affiliations:** 1 New York University Medical Center, New York, NY, USA; 2 University of Massachusetts Medical School, Worcester, MA, USA

## Background

The rabbit is a commonly used animal model to study antibody responses in AIDS vaccine development. However, little is known about the relationship between epitopes recognized by the rabbit and human immune systems. Structural knowledge of antigen-antibody interactions of rabbit and human mAbs will help us understand the similarity of these two immune systems in recognizing HIV antigens, thus provides a guidance in using the rabbit for AIDS vaccine development.

## Methods

Complex structures of anti-V3 mAbs R56 and R20, generated by immunizing a rabbit with JR-FL gp120 using a DNA prime-protein boost regimen, were determined and analyzed in comparison with human mAbs from HIV-1 infected patients against the same V3 immunogenic regions.

## Results

The epitope of R56 is structurally mapped to the N-terminal region of the V3 crown, overlapping with the epitopes recognized by human IGHV5-51 germline anti-V3 mAbs. Both R56 and the human mAbs bind the highly conserved V3 residues, consistent with their broad neutralization activities. However, while the human antibodies can bind the whole beta-hairpin of the crown, R56 only binds the N-terminal half of the hairpin. The epitope of R20 is located in the V3 C-terminal region near the two highly conserved glycosylation sites at the V3 base. This epitope overlaps with that of human mAb PGT128. A long beta-hairpin CDR H3 of R20 stands at the center of its antigen-binding site in a manner similar to several potent human mAbs such as 2909, and interacts with the epitope by a beta-sheet-type interaction.

## Conclusion

Structural analyses of immunization-generated rabbit antibodies show that they can recognize immunogenic regions of gp120 and mimic the binding modes of human antibodies. However, optimized immunization schemes need be tested in rabbits to produce antibodies with sufficient affinity maturation to recognize Env epitopes as complex as that of human antibodies generated in chronic infected patients.

